# 12-Mo Intervention of Physical Exercise Improved Work Ability, Especially in Subjects with Low Baseline Work Ability

**DOI:** 10.3390/ijerph110403859

**Published:** 2014-04-04

**Authors:** Oili Kettunen, Timo Vuorimaa, Tommi Vasankari

**Affiliations:** 1 Department of Health and Exercise & Paavo Nurmi Center, University of Turku, 20520 Turku, Finland; 2 Department of Health and Exercise, Sports Institute of Finland, 19120 Vierumäki, Finland; 3 Haaga-Helia the University of Applied Sciences, 19120 Vierumäki, Finland; E-Mail: timo.vuorimaa@haaga-helia.fi; 4 UKK Institute for Health Promotion Research, 33501 Tampere, Finland; E-Mail: tommi.vasankari@uta.fi; 5 National Institute for Health and Welfare, 00271 Helsinki, Finland

**Keywords:** cardiorespiratory fitness, exercise, work ability

## Abstract

*Objectives*: This study’s objective was to assess the effects of a 12-month physical exercise intervention on work ability (WAI) and cardiorespiratory fitness (CRF) in healthy working adults. *Methods*: The study group had 371 participants, of which 338 (212 women and 126 men) were allocated in the exercise group and 33 (17 women and 16 men) in the control group. The exercise group underwent a 12-month exercise program followed by a 12-month follow-up. WAI and CRF were evaluated at baseline, and at 4, 8, 12, and 24 study months, in both exercise and control groups. The exercise group was divided into subgroups according to baseline WAI classifications (poor/moderate, good, excellent). *Results*: During the 12-month exercise intervention, the exercise group increased their leisure-time physical activity by 71% (*p* = 0.016) and improved the mean WAI by 3% and CRF by 7% (*p* < 0.0001, in both), while WAI and CRF decreased in the control group (ANCOVA using age, sex and BMI as covariates, for WAI, *p* = 0.013 and for CRF, *p* = 0.008). The changes in WAI and CRF between the exercise group and control group were significantly different during the intervention (baseline *vs*. 12-months, *p* = 0.028 and *p* = 0.007) and after the follow-up (*p* = 0.001 and *p* = 0.040), respectively. A light positive correlation between the changes in WAI and in CRF (*r* = 0.19, *p* < 0.01) existed. WAI improvement was the highest (13%, *p* < 0.0001) in the subgroup having poor/moderate WAI at baseline (ANCOVA, *p* < 0.001). *Conclusions*: The improvement of WAI associated with CRF. These results suggest that a physical exercise intervention may improve work ability.

## 1. Introduction

Physical activity is one option to increase work ability [1]. In a systematic review of 14 cross-sectional studies and six longitudinal studies assessing the effects of work-related and individual factors on the Work Ability Index (WAI), the factors associated with the poor work ability were: lack of leisure time, vigorous physical activity, poor musculoskeletal capacity, older age, obesity, high mental work demands, lack of autonomy, poor physical work environment, and a high physical work load [2]. However, the relationship between increased physical activity and improved fitness on perceived work ability is inconsistent [3,4]. Therefore, even if physical exercise interventions are commonly used in promoting employees’ physical and psychosocial functioning and work ability, limited scientific evidence about the effectiveness of such programs exist [5,6,7,8,9,10]. The long-term effects of exercise interventions on WAI are needed [11].

The WAI, developed by the Finnish Institute of Occupational Health, is a questionnaire-based method, which assesses perceived work ability [12]. WAI is widely used, and a validated instrument to measure work ability [13]. The work ability concept is based on the assumption that work ability is determined by persons` perceptions of the demands on them and their ability to cope with the demands. WAI describes the balance between work demands and a person`s functional capacity (e.g., health, physical capacity, competence, values) [14] and physical activity interventions are suggested to improve work ability in both skilled and unskilled workers.

In this study, we investigated the effects of a 12-month exercise-training program with moderate volume and low to moderate intensity on WAI responses in healthy working adults. We hypothesized that exercise intervention causes increased cardiorespiratory fitness and improves work ability. WAI and cardiorespiratory fitness were measured before and after: 4-, 8- and 12-month exercise training programs and after a 12-month follow-up.

## 2. Methods

The study group comprised of 371 employees, whom were recruited from small and medium-sized companies (less than 100 employees per company) with the help of local entrepreneur associations and occupational health care centers in the Päijät-Häme region of Southern Finland. The study protocol was approved by the ethical committee of the Pirkanmaa hospital district. The inclusion criteria were: healthy, no permanent medication, an age range of 20–60 years (mean age: women, 44/men, 42 years), and no contraindications to walking exercise (Table 1). The participants were divided into exercise and control groups, such that about 10 percent of the participants would comprise the control group. The controls were from the same companies as the intervention participants, but they came from a different department, which separated them from the intervention. Participants in the control group were selected such that there would be approximately as many men as women and an even difference in skilled and unskilled workers. They were a similar age as the exercise group and that their leisure time physical activity (LTPA) at baseline did not differ from that of exercise group. The exercise group consisted of 338 participants (85 unskilled and 253 skilled workers consisting of 212 women and 126 men) and the controls had 33 subjects (19 unskilled and 14 skilled; 17 women and 16 men). No differences were reported in the level of education between the exercise group and control group. The mean distance to work was 13.4 km and most participants used their car (83%). Only 17 percent of the participants walked or bicycled to work.

**Table 1:** Participant characteristics of the exercise intervention group (*n* = 338) and control group (*n* = 33). Mean (SD), or %.

Characteristic	Exercise group mean (± SD) *n* = 338	Control group mean (± SD) *n* = 33	*p*-value exercise *vs.* control
Women/men	212/126	17/16	
Age (y)	45 (8.8)	41 (6.9)	0.357 ^a^
Weight (kg)	76 (15.3)	81 (12.6)	0.104 ^a^
Height (cm)	170 (8.7)	169 (8.3)	0.403 ^a^
BMI	26.1 (4.4)	27.4 (4.0)	0.693 ^a^
MET/h/wk	8.2 (8.7)	7.7 (10.1)	0.398 ^a^
Use of alcohol/doses per week	7.7 (10.5)	8.7 (11.0)	0.565 ^b^
Current smoker (%)	21.0	12.0	0.816 ^b^
Level of education (%)			0.171 ^b^
Education low	38.0	50.0	
Education moderate	40.0	20.0	
Education high	22.0	30.0	

^a^ Independent samples t-test; ^b^ Chi Square test.

### 2.1. Study Design

The exercise group underwent a 12-month exercise program, which contained two-day training camps at the Sport Institute of Finland, at baseline, 4-, 8-, 12- and 24-months. The training camps contained measurements (weight, height, fat %, a bicycle ergometer test, muscle- and flexibility tests, and questionnaires). The participants’ exercise regimen was supervised and given lessons about health- related issues, such as physical fitness and muscle care. The persons in the exercise group formed groups of 15 to 20 persons within the same company and each group had a coach (physiotherapist or exercise instructor). During the whole intervention, the same coach guided the specific groups.

Between training camps, the exercise program contained one to two supervised exercise sessions in a group per month and three to five unsupervised exercise sessions per week (mainly walking, skiing and biking). Every participant had also an individualized exercise program based on their estimated oxygen uptake (VO_2max_). During the exercise, the mean heart rate was mainly at the moderate level, mainly 60–80 percent of the participant’s estimated maximal heart rate. Exercise intensity was monitored with Polar heart rate microcomputers (Polar Electro, Kempele, Finland). Exercise amount and intensity between training camps were recorded with personal, web-based, exercise diaries. The coach viewed, twice-a-month, the content of the exercise diary and gave more specific information if needed concerning the exercise programs for the following weeks. The control group had no supervised exercise or program. After 12-months of the supervised exercise program, there was a 12-month follow-up without exercise coaching, to evaluate the possible stability or relapse of the results of the intervention.

### 2.2. Questionnaires

Work ability was measured with the WAI [12]. WAI is a questionnaire-based method for use in occupational health care and researches consisting of seven indicators that gauge their well-being and, in addition, how capable are they to do their work with respect to their work demands, health, and mental resources [14,15]. In addition to the subjective estimations of work ability in relation to work demands, WAI also includes the dimensions of the determinants (*i.e.*, health) and outcomes (*i.e.*, consequences of health, in terms of sick leave and functional limitations) of work ability. The WAI is translated into 26 languages and is used in several countries [16] and the reliability and validity of WAI is well documented [17]. The test-retest reliability of the mean WAI-score and classification into WAI categories were found to be a stable measure over a four-week interval among 97 elderly construction workers. Exactly the same WAI score on both measurements was reported in 25 percent of the participants and 95 percent of the individual differences between measurements were less than 6.86 points (two times the standard deviation) [18].

The sum score forms the WAI, which is classified into the following four categories of work ability: 1. poor (values: 7–27), 2. moderate (values: 28–36), 3. good (values: 37–43), and 4. excellent work ability (values: 44–49) [14]. At baseline, participants were categorized into four work ability classes according to the work ability index and for the statistical analysis, the two lowest WAI groups were combined: WAI scores of seven to 36 (*n* = 43) were poor/moderate, the WAI scores of 38–43 (*n* = 168) were good, and WAI 44–49 (*n* = 127) excellent.

### 2.3. Life Style Questionnaire

Use of alcohol, tobacco, and leisure time physical activity habits were asked at baseline and after 12-months with a questionnaire. The exercise amount and intensity between training camps was also evaluated with personal exercise diaries *via* the internet. The weekly LTPA was determined using three questions concerning frequency, duration and intensity of the exercise [19]. According to the answers, we calculated the metabolic equivalent (MET) h/week values (LTPA times per week x duration x intensity) (Table 1). One MET represents the approximate rate of VO_2_ consumption of a seated individual at rest, which is 3.5 mL O_2_/kg/min [10,20]. The distance and the method of travelling to work were also asked.

### 2.4. Cardiorespiratory Fitness (VO_2max_)

Maximal oxygen uptake was extrapolated indirectly on the basis of an electrically braked bicycle ergometer (Ergoline 800S, Ergoline, Berlin, Germany) test (pedal rate, 60 rpm) using three submaximal loads. The lowest submaximal load was calculated to produce a heart rate of approximately 120 beats per minute and the highest submaximal load approximately 85 percent of age-adjusted maximal heart rate. The age-adjusted maximal heart rate was derived from the reference values of Seliger and Bartunek [21], and maximal oxygen uptake was extrapolated from the maximal heart rate. The same individual workloads that were used at the baseline test were used during the follow-up tests. Lower heart rates at the highest load prompted the additional fourth work load to increase the heart rates to the expected levels.

### 2.5. Statistical Analyses

Statistical analyses were run by SPSS (Statistical Package for Social Sciences, version 19.0, IBM (Internalional Business Machines, New York, NY, USA) software. The normality of the variables was tested and logarithm transformations were applied in case of abnormal distributions. After the test of normality, repeated measures ANOVA was first done for the intervention and control groups. Trial * group (intervention/control) interaction in repeated measures ANOVA was used separately for the intervention (baseline/4-mo/8-mo/12-mo) and follow-up (baseline/24-mo) samples. With a significant ANOVA, age, gender, and BMI were used as covariates (ANCOVA). A paired t-test within the intervention or control group was done as a *post-hoc* test. Similarly, the difference among subgroups was analyzed by ANOVA and ANCOVA using Bonferroni`s correction as a *post-hoc* test. We used Pearson’s correlation to evaluate the correlations. First we calculated the difference of change between baseline and 12-months in both CRF, LTPA and WAI. Thereafter, the difference of change seen in WAI was correlated to the difference of change in CRF and LTPA. There were no significant differences in results between genders or skilled/unskilled workers, therefore the results were combined.

## 3. Results

The number of participants in the exercise intervention and the measurement of the exercise group were: at baseline, 338 participants; at four-months, *n* = 276 (82%); at eight-months, *n* = 306 (91%); at 12-months, *n* = 306 (91%); and at 24-months, *n* = 178 (53%). Participation in the measurements of the control group was: at baseline, *n* = 33; at four-months, *n* = 29 (88%); at eight-months, *n* = 27 (82%); at 12-months, *n* = 28 (85%); and at 24-months, *n* = 28 (85%). At baseline, 23 percent of the exercise group were current smokers and there were no changes in smoking or alcohol use habits during the intervention (Table 1).

### 3.1. Work Ability Index (WAI)

During the 12-month intervention, the WAI score improved after four-months by two percent (*p* = 0.001), after eight-months by three percent (*p* < 0.001), after 12-months by three percent (*p* < 0.001) in the exercise group (Table 2). In the control group, WAI decreased at 12-months by two percent (*p* = 0.066) and between baseline and 24-months, by four percent (*p* = 0.003).

The subgroup, having poor/moderate WAI score at baseline, improved their WAI the most. After four-months by nine percent (*p* < 0.001), after eight-months by 11% (*p* < 0.001), after 12-months by 13% (*p* < 0.001). After the 12-month follow-up, WAI was still 11% (*p* = 0.024) improved.

**Table 2:** Work ability index (WAI) and Vo_2_max (ml/kg/min) trial * group (intervention/control) interaction in exercise intervention group (*n* = 338) and control group (*n* = 33), during exercise intervention (baseline (0 months), 4-, 8- and 12-months) and follow-up (24-months). The ANCOVA model contains age, sex, and BMI as covariates.

Measurement	Baseline	4-mo	8-mo	12-mo	24-mo		ANOVA during intervention 0–12-mo	ANCOVA during intervention 0–12-mo	ANOVA after follow-up 0–24-mo	ANCOVA after follow-up 0–24-mo
WAI										
exercise	41.4(4.7)	42.1(4.5) **	42.7(4.5) ***	42.8(4.6) ***		42.0(4.9)	*p* = 0.002	*p* = 0.013	*p* = 0.011	*p* = 0.021
control	42.5(4.9)	42.5(4.6)	42.5(5.1)	41.7(4.8)		40.7(5.3)				
Vo_2_max(ml/kg/min)										
exercise	32.1(0.7)	33.4(8.0) ***	33.9(8.1) ***	34.4(7.6) ***		33.6(7.7) **	*p* = 0.005	*p* = 0.008	*p* = 0.019	*p* = 0.015
control	35.4(11.3)	35.7(10.8)	35.5(12.0)	34.4(10.9)		34.6(11.2)				

Differences in exercise intervention group and control group from baseline; * 0.01< *p* ≤0.05; ** *p* <0.01; *** *p* <0.001.

The subgroup had a WAI score, classified as good at baseline, and improved their WAI after four-months by three percent (*p* < 0.001), after eight-months by five percent (*p* < 0.001), after 12-months by five percent (*p* < 0.001). After the 12-month follow-up, WAI remained elevated by three percent (*p* = 0.025) (Table 3). The change in WAI between the exercise group and the control group, during exercise intervention, (baseline *vs.* 12-months) was *p* = 0.028 and after follow up (baseline *vs.* 24-months) was *p* = 0.001.

**Table 3:** Work ability index (WAI) subgroups during intervention (baseline, 4-, 8- and 12-months) and follow-up (24-mo). Mean (SD). The ANCOVA model contains age, sex and BMI as covariates.

		0-mo	4-mo	8-mo	12-mo	24-mo	
WAI excellent		45.7(1.5)	45.1(2.9)	45.4(3.1)	45.6(2.7)	44.8(2,8)	
*n* = 127							
WAI good		40.2(1.9)	41.5(3.3) ***	42.1(3.5) ***	42.2(3.6) ***	41.4(4.0) **	
*n* = 168							
WAI poor/moderate		32.6(3.6)	35.5(4.3) **	36.1(5.1) ***	37.0(6.3) ***	36,1(5,2) **	
*n* = 43							

ANOVA/ANCOVA: 0-12-mo, *p* < 0.0001; ANOVA/ANCOVA: 0- *vs.* 24-mo, *p* < 0.0001; Difference within subgroups from 0-mo:* 0.01< *p* ≤ 0.05; ** 0.001< *p* ≤ 0.01; *** *p* < 0.001.

### 3.2. Cardiorespiratory Fitness (VO_2max_ (mL/kg/min))

The VO_2max_ improved in the exercise group after four-months by four percent (*p* < 0.0001), after eight-months by six percent (*p* < 0.001) and after 12-months by seven percent (*p* < 0.001) and from baseline to 24-months by five percent (*p* < 0.001, Table 2), while VO_2max_ decreased three percent after 12 months in the control group. There was a significant correlation between the change in WAI and the change in VO_2max_ (mL/kg/min) (*r* = 0.19, *p* < 0.01, *n* = 335). The change in cardiorespiratory fitness between exercise group and control group during intervention (baseline *vs*. 12-month) was *p* = 0.007 and after follow-up (baseline *vs.* 24-month) was *p* = 0.040.

### 3.3. Leisure Time Physical Activity (LTPA)

According to the internet-based exercise diaries (*n* = 209), the mean LTPA during active year was 19.5 (SD: 11.0) MET hours/week and during the follow-up year, 15.7 (SD: 12.3) MET/h/wk. According to the questionnaire (*n* = 338), the exercise group increased their self-reported LTPA, during the intervention, by 71 percent (*p* = 0.016). The mean (SD) of LTPA increased from 8.3 (8.8) MET h/wk at baseline was 14.2 (SD: 47.2) MET/h/wk to 12-month in the exercise group, but the increase of LTPA did not correlate with the change in WAI (*r* = 0.05, *p* = 0.33). The mean (SD) of LTPA of the control group was at baseline 7.7 (10.1) MET/h/wk and at 12-months, 7.9 (10.2) MET/h/wk.

## 4. Discussion

In this study, we investigated the effect of a 12-month physical exercise intervention and a 12-month follow-up on the work ability and cardiorespiratory fitness in working adults. During the 12-month exercise intervention, the mean WAI improved by three percent and CRF by seven percent in the exercise group, while the WAI and CRF tended to decrease in the control group. A trend for a positive correlation existed between the change in WAI and the change in CRF.

WAI is used in exercise intervention studies and improved physical fitness does not automatically lead to enhanced work ability [3]. WAI scores did not improve simultaneously with cardiovascular fitness in a 6-month “Project active” [3]. Similarly, enhanced CRF does not relate with improved WAI among home helpers (*n* = 70) [22] and police officers (*n* = 80) [23]. The difference in results between the present study and earlier studies may be caused by a greater number of the participants and longer study period.

Our results indicate that poor WAI can be improved with an exercise intervention that includes increased cardiorespiratory fitness. The amount of LTPA increased significantly in the exercise group, but there was no significant correlation between the change in LTPA and the change in WAI score. In an eight-month intervention study with 111 policemen, no improvement occurred in WAI, although the physical activity and fitness increased [24]. However, the increased amount of vigorous LTPA was one significant factor explaining the improvement of WAI in an 11-year follow-up study in 6,259 older municipal workers [25]. But LTPA does not associate with work ability among office workers [26].

This study design was effective because participant number were high, we used several time points of measurements, and had a long span. However, study also has some limitations. Because the study protocol was set in a “real-life” work life, we decided not to use randomization and allocated only 10 percent of participants to control group. The amount of drop-outs was quite low during the intervention period, but somewhat higher than expected during the follow-up year. A call-up letter was sent to the drop-outs and a lack of compliance in the follow-up measurement was most likely due to retirement, changing the employer, sickness, and other personal reasons. It is well known that non-randomized trials may have methodological problems. Therefore, we tried to diminish this by using high number of participants from several different companies, several (4) time points of measurements during the intervention, one year follow-up and different methods to evaluate the physical activity/fitness (questionnaire, exercise diary, fitness tests five times.

The average WAI score at baseline was good (41 points) and the prevalence of lowered work ability (poor/moderate WAI score) was 13 percent for the study sample. Our result is in line with previous studies, where 15 percent of the employees generally have the lowered work ability [27]. The average WAI score in the lowest work ability group at baseline (*n* = 43) improved gradually from an average of 32 to 37 during the intervention, which implies that the mean result improved from the moderate work ability category to the good category. Importantly, the subgroup that benefitted the most from the exercise intervention was the group that had the lowest WAI at baseline. There were significant differences in changes in WAI score and changes in cardiorespiratory fitness between the exercise and control groups, during the intervention period and after the follow-up year. It cannot be ruled out, that additional factors could have improved the work environment and work ability, but it is very improbable that other confounding factors occurred simultaneously at many different companies. According to the questionnaires, participants increased their LTPA by 71 percent during the intervention. Both individual exercise program and supervised exercise sessions were planned to contain enough aerobic physical activity, which could lead to increased cardiorespiratory fitness and would therefore improve WAI. The participants also maintained internet-based exercise diaries, which should enhance the adherence to the intervention.

Exercise intervention was effective and this kind of exercise intervention could be recommended for future use targeting, especially, employees with low baseline workability. Targeting employees at high risk and great need is a challenge for campaigns of employee’s well-being [28]. Important factors associated with a poor work ability is demonstrated to be lack of leisure-time vigorous physical activity, poor musculoskeletal capacity, older age, obesity, high mental work demands, lack of autonomy, poor physical work environment, and high physical work load [2]. Identifying employees at with these risks is a challenge for employers.

## 5. Conclusions

In conclusion, our results suggest that a physical exercise intervention that results in enhanced cardiorespiratory fitness may improve work ability. The WAI improved especially in the subjects with baseline poor to moderate work ability. The improved WAI was associated with improved cardiorespiratory fitness.
